# Perioperative Management of a Spinal Cord Stimulator During Transcatheter Aortic Valve Implantation: A Case Report

**DOI:** 10.7759/cureus.118692

**Published:** 2026-10-27

**Authors:** Jonathan Zheng, Julian Yeoh, Matias Yudi, Anoop N Koshy, Laurence Weinberg

**Affiliations:** 1 Department of Anaesthesia, Austin Health, Heidelberg, AUS; 2 Department of Cardiology, Austin Health, Heidelberg, AUS; 3 Department of Critical Care, The University of Melbourne, Melbourne, AUS

**Keywords:** anaesthesia, case report, defibrillation, electromagnetic interference, neuromodulation, rapid ventricular pacing, spinal cord stimulation, transcatheter aortic valve implantation

## Abstract

Spinal cord stimulation (SCS) is an established treatment for chronic neuropathic pain, and patients with an implanted stimulator are presenting with increasing frequency for transcatheter aortic valve implantation (TAVI). We describe an 88-year-old man with a high-frequency (10 kHz) spinal cord stimulator for chronic back pain who underwent transfemoral TAVI under sedation for severe symptomatic aortic stenosis. After consultation with the implanting pain service, the stimulator was switched off with the patient's own remote control, which was kept in the procedure room; rapid ventricular pacing was delivered through a bipolar right ventricular lead so that the pacing current was confined to the myocardium and remote from the pulse generator; and external defibrillator pads were positioned away from the generator and the course of the leads. The procedure was uneventful. The patient tolerated the interruption of therapy without clinically significant pain; a new left bundle branch block after valve deployment was identified on an artefact-free trace, stimulation was resumed after the procedure with analgesia equivalent to baseline, and subsequent interrogation confirmed normal device function. The case illustrates the application of a precautionary strategy informed by manufacturer labelling and analogous evidence; because the stimulator was off throughout, it does not establish that any individual measure was necessary. We examine how the hazards of the cardiac catheterisation laboratory differ from those of the operating theatre and propose a structured six-step approach to the management of a spinal cord stimulator during TAVI.

## Introduction

Spinal cord stimulation (SCS) is an established treatment for chronic neuropathic pain that has proved refractory to conservative and pharmacological therapy, and its use continues to expand [1,2]. The therapy has also diversified. Conventional paraesthesia-based stimulation has been joined by high-frequency (10 kHz), burst, and closed-loop paradigms whose mechanisms of action remain incompletely understood [3,4], and contemporary systems differ between manufacturers in their programming, magnetic resonance conditionality, and dedicated surgical modes [5]. Because chronic pain affects more than one in three adults aged 65 years and older [6], an increasing proportion of patients with a spinal cord stimulator in situ are elderly, and many will require cardiovascular intervention during the working life of their device.

Transcatheter aortic valve implantation (TAVI) is among the cardiovascular interventions that such patients may require. Procedural volumes continue to rise [7,8], and practice has moved away from general anaesthesia: conscious sedation was used in 64% of transfemoral procedures in the Society of Thoracic Surgeons-American College of Cardiology Transcatheter Valve Therapy (STS-ACC TVT) Registry by 2019 [9], and randomised data have confirmed that local anaesthesia with sedation is not inferior to general anaesthesia [8]. The typical TAVI recipient, an octogenarian with degenerative disease and often a long history of musculoskeletal pain, is therefore a patient in whom a spinal cord stimulator may be encountered.

Published guidance on the perioperative management of a spinal cord stimulator has been written for the operating theatre [5,10-12]. Its recommendations are consistent: identify the device and its manufacturer, reduce the amplitude and switch the stimulator off before induction, avoid monopolar diathermy or, where it is unavoidable, place the return electrode remote from and contralateral to the pulse generator, and confirm device function afterwards [5,10]. These recommendations rest on three assumptions: a general anaesthetic, a surgical wound, and electrosurgery as the principal source of electromagnetic interference. None holds for percutaneous transfemoral TAVI. The patient is usually awake or lightly sedated and must lie still through valve crossing and deployment; diathermy is seldom used; and the relevant electrical exposures are instead rapid ventricular pacing, typically at 160-220 beats per minute through a temporary transvenous or left ventricular guidewire electrode [13], the possibility of external defibrillation or cardioversion during a catastrophic intraprocedural event, which occurred in 2.5% of 2,102 procedures in one large single-centre series [14], and the small but persisting risk of conversion to emergent open surgery, 0.76% in a European registry of 27,760 transfemoral procedures [15].

Continuous electrocardiographic surveillance is central to periprocedural safety, since new left bundle branch block and high-grade atrioventricular block remain the commonest complications of TAVI and permanent pacemaker implantation follows in roughly one patient in 10 [7,16]. An active neurostimulator can degrade the surface electrocardiogram [17], and although interactions between SCS systems and cardiac implantable electronic devices appear to be uncommon [18,19], no guidance addresses the transient coexistence of an SCS with a temporary pacing system during structural heart intervention.

To our knowledge, only one previous report mentions a spinal cord stimulator during TAVI, noting that the device was placed in Surgery Mode before an uneventful procedure, without describing its periprocedural management [20]. We describe the periprocedural management of an 88-year-old man with a high-frequency spinal cord stimulator who underwent transfemoral TAVI under sedation for severe aortic stenosis, examine how the hazard profile of the cardiac catheterisation laboratory differs from that of the operating theatre, and propose a structured approach to device management in this setting. The report has been prepared in accordance with the CARE guidelines [21].

## Case presentation

Presentation

An 88-year-old man was admitted to the cardiology unit for expedited transfemoral TAVI for severe symptomatic aortic stenosis. The stenosis had been diagnosed a year earlier and was initially asymptomatic, but over the preceding months, he had developed progressive exertional dyspnoea, paroxysmal nocturnal dyspnoea, and exertional chest discomfort, with an exercise tolerance of approximately 200 metres. On day 1, he was admitted to another hospital after a fall complicated by a subdural haematoma, which was managed conservatively. During that admission, he experienced a 15-minute episode of central chest pain at rest, with a peak high-sensitivity troponin of 46 ng/L (normal range: 0-21 ng/L in males) and new electrocardiographic changes, and on day 40, he was transferred to our cardiology unit for assessment for TAVI. The resting electrocardiogram showed sinus rhythm with first-degree atrioventricular block (PR interval: 229 ms), and transthoracic echocardiography demonstrated concentric left ventricular wall thickening (Figure 1).

**Figure 1:**
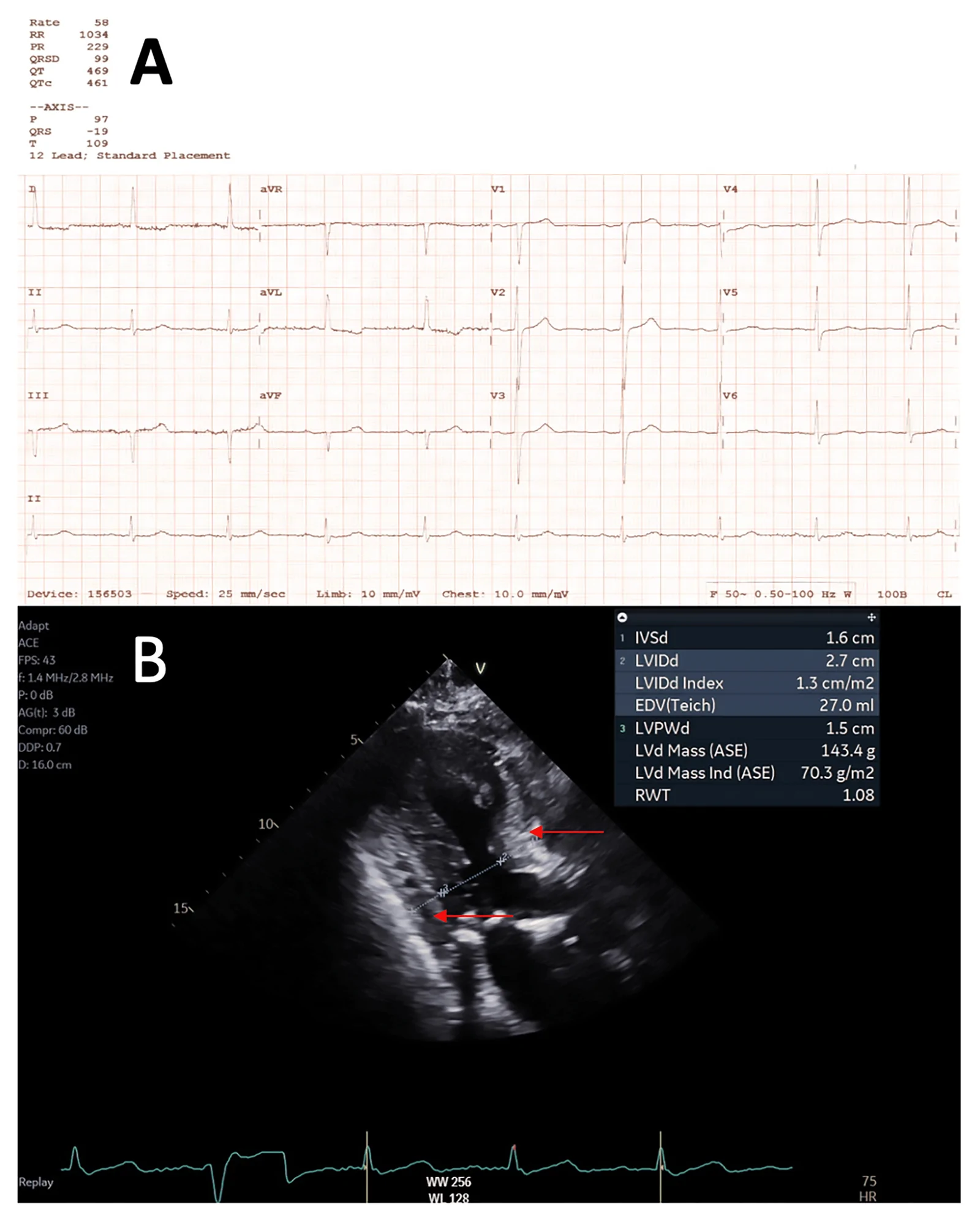
Twelve-lead electrocardiogram and two-dimensional transthoracic echocardiography. (A) Sinus rhythm with first-degree atrioventricular block. The automated measurement panel (top left) showing a ventricular rate of 58 beats per minute, RR interval of 1034 ms, PR interval of 229 ms, QRS duration of 99 ms, QT interval of 469 ms, and corrected QT interval of 461 ms, with a P wave axis of 97 degrees, QRS axis of minus 19 degrees, and T wave axis of 109 degrees, recorded at 25 mm/s and 10 mm/mV with a 0.50-100 Hz filter. (B) Parasternal long-axis view demonstrating thickening of the interventricular septum (upper arrow) and left ventricular posterior wall (lower arrow), with a RWT of 1.08. PR: PR interval; QRSD: QRS duration; QT: QT interval; QTc: corrected QT interval; RR: RR interval; IVSd: interventricular septal thickness at end diastole; LVIDd: left ventricular internal diameter at end diastole; LVIDd Index: LVIDd indexed to body surface area; EDV (Teich): left ventricular end-diastolic volume estimated using the Teichholz method; LVPWd: left ventricular posterior wall thickness at end diastole; LVd Mass (ASE): left ventricular mass calculated from end-diastolic measurements using the American Society of Echocardiography (ASE) linear formula [22]; LVd Mass Ind (ASE): calculated left ventricular mass indexed to body surface area; RWT: relative wall thickness, calculated as twice LVPWd divided by LVIDd (dimensionless); HR: heart rate; WL: window level; WW: window width

Transthoracic echocardiography demonstrated a calcified trileaflet aortic valve, and continuous-wave Doppler interrogation confirmed severe aortic stenosis (Figure 2).

**Figure 2:**
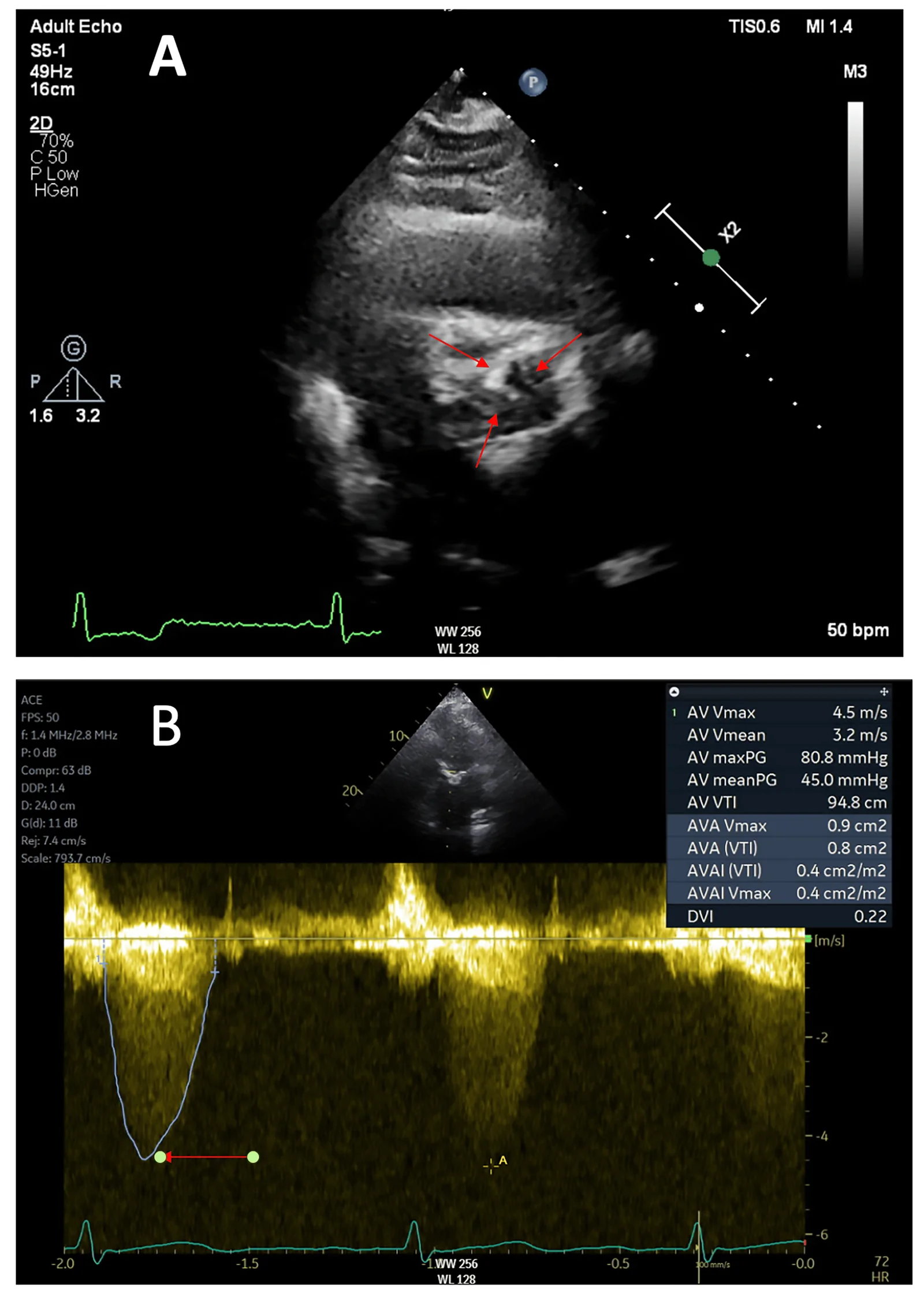
Transthoracic echocardiography of the aortic valve. Parasternal short-axis view at the level of the AV demonstrating a trileaflet valve with marked leaflet thickening and prominent echogenic calcific deposits affecting the right coronary, left coronary, and non-coronary cusps (arrows, from top to bottom) (A) and continuous-wave Doppler spectral display obtained from the apical window, with the two-dimensional reference image shown above the spectral trace (B). The high-velocity systolic jet is displayed below the baseline, directed away from the transducer. The traced envelope (blue) of a representative beat shows a peak transaortic velocity of 4.5 m/s (arrow), mean velocity of 3.2 m/s, peak gradient of 80.8 mmHg, mean gradient of 45.0 mmHg, and velocity time integral of 94.8 cm. The aortic valve area was 0.8 cm² by the velocity time integral method and 0.9 cm² by the peak velocity method (0.4 cm²/m² when indexed to body surface area), with a DVI of 0.22. These values meet guideline criteria for severe aortic stenosis (peak velocity ≥4.0 m/s, mean gradient ≥40 mmHg, and aortic valve area <1.0 cm²) [23]. The simultaneous electrocardiogram is displayed at the bottom of the trace (HR 72 bpm). Velocity scale in m/s; sweep speed 100 mm/s. AV: aortic valve; AVA: aortic valve area; AVAI: aortic valve area indexed to body surface area; DVI: Doppler velocity index; HR: heart rate; maxPG: peak pressure gradient; meanPG: mean pressure gradient; Vmax: peak velocity; Vmean: mean velocity; VTI: velocity time integral; WL: window level; WW: window width

The left ventricular ejection fraction was preserved (Figure 3).

**Figure 3:**
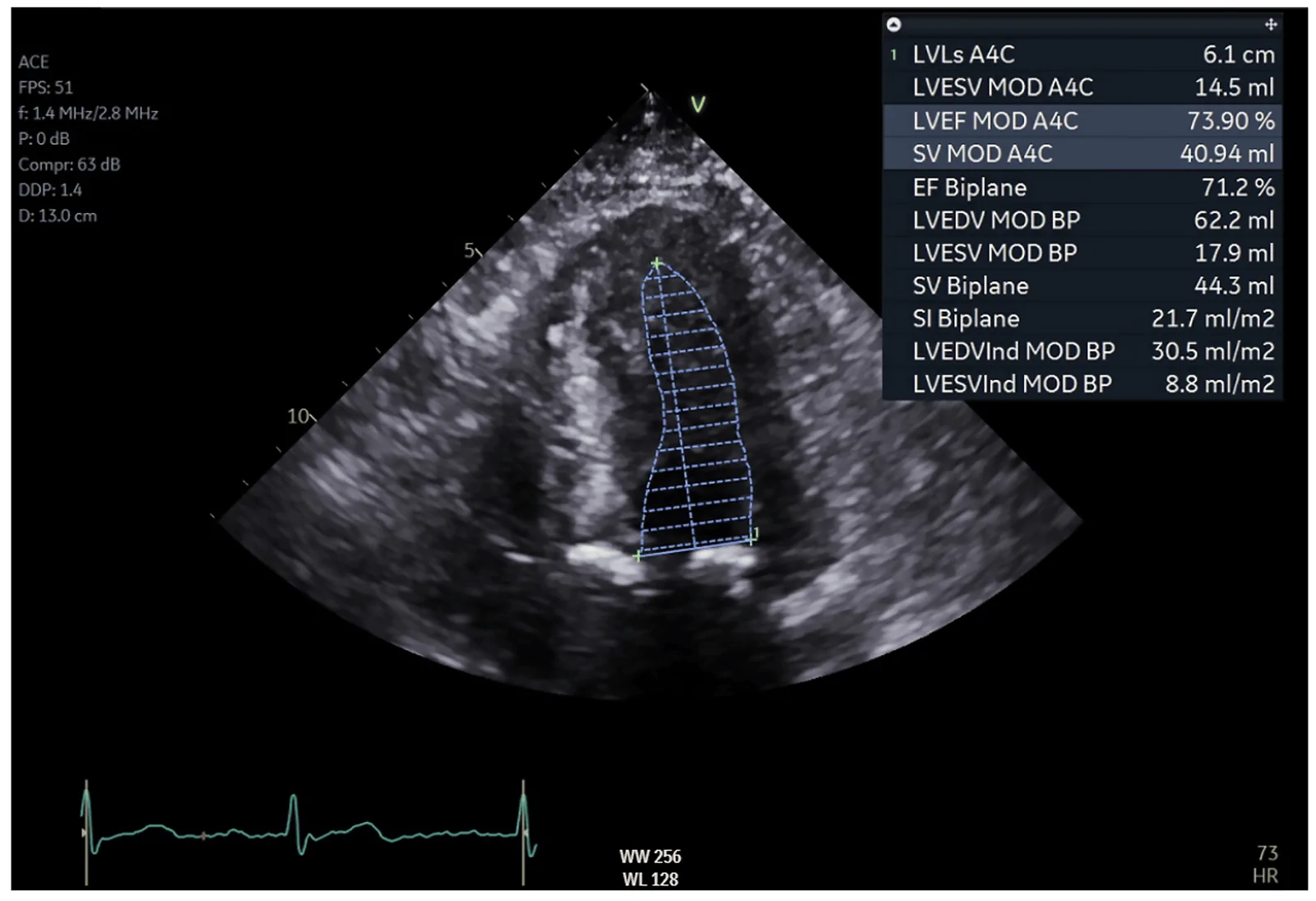
A4C view at end systole demonstrating preserved left ventricular systolic function. The left ventricular endocardial border has been traced (blue) for volumetric quantification by the MOD (modified Simpson's rule). The measurement panel (top right) shows a single-plane A4C LVEF of 73.9% and a biplane EF of 71.2% (highlighted), with LVEDV of 62.2 mL, LVESV of 17.9 mL, SV of 44.3 mL, SI of 21.7 mL/m², and body-surface-area-indexed volumes of 30.5 mL/m² (LVEDVInd) and 8.8 mL/m² (LVESVInd). The red marker on the simultaneous ECG indicates the end-systolic frame; heart rate 73 beats/min. The left ventricular walls appear thickened with a small end-systolic cavity [22]. A4C: apical four-chamber; MOD: method of discs; LVEF: left ventricular ejection fraction; EF: ejection fraction; LVEDV: left ventricular end-diastolic volume; LVESV: left ventricular end-systolic volume; SV: stroke volume; SI: stroke index: ECG: electrocardiogram

After cardiology assessment, the troponin rise was attributed to demand ischaemia in the setting of severe aortic stenosis rather than to an acute coronary event. Following heart team review, he was accepted for transfemoral TAVI and discharged, with the procedure scheduled for day 43 so that six weeks would elapse between the subdural haematoma and exposure to intraprocedural heparin.

Medical history and the neuromodulation system

His history was notable for chronic back pain treated with a spinal cord stimulator implanted eight years earlier, which had provided sustained and effective analgesia. Other comorbidities were hydrocephalus treated with a right fixed-pressure ventriculoperitoneal shunt, Parkinson's disease, ischaemic heart disease, hypertension, dyslipidaemia, chronic kidney disease (estimated glomerular filtration rate 61 mL/min/1.73 m²), asthma, psoriatic arthritis, gout, a left total knee replacement, septic arthritis of the right native knee, osteomyelitis of the left second toe, gastro-oesophageal reflux disease, and an overactive bladder managed with a long-term indwelling urinary catheter. Vital signs at the pre-anaesthetic assessment were within normal limits. Airway examination, undertaken in case conversion to general anaesthesia became necessary, revealed no predictors of a difficult airway other than cervical extension limited by pain, which was not accompanied by upper limb neurological symptoms.

At the pre-anaesthetic assessment on day 42, the implanting pain service was contacted, and its clinical technician confirmed the device details: a high-frequency (10 kHz) Senza HFX system (Nevro Corp., Redwood City, CA, USA) with an NIPG1500 implantable pulse generator sited subcutaneously in the left lower back, controlled by a handheld remote that the patient carried and could operate himself. Plain radiography confirmed two percutaneous epidural leads with their electrode contacts over the lower thoracic spine, at the level of T8-T11 (Figure 4), and a frontal computed tomography scout image showed the pulse generator in the left lower back and gluteal region (Figure 5).

**Figure 4:**
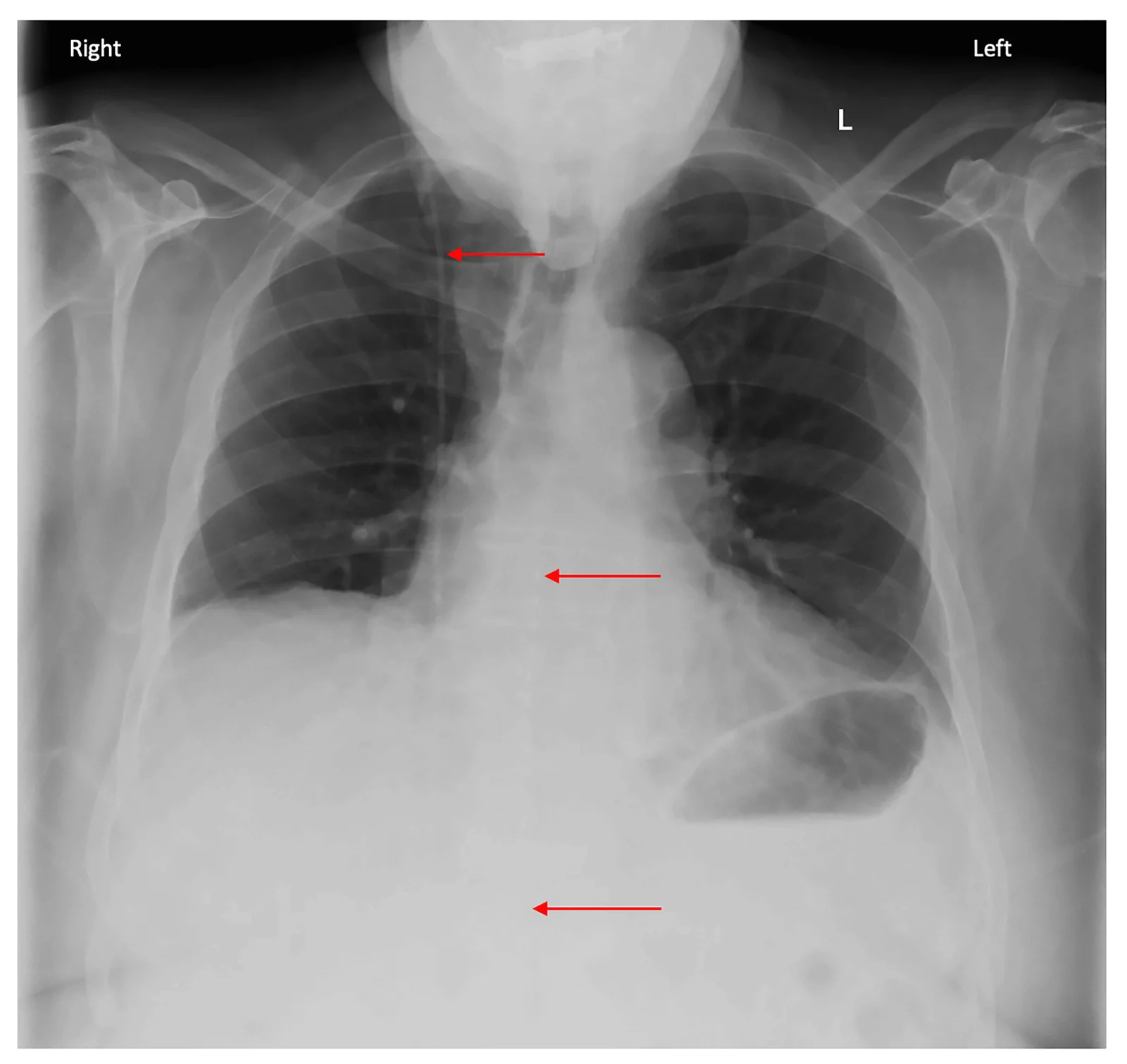
Frontal chest radiograph showing the ventriculoperitoneal shunt tubing and the thoracic epidural leads of the spinal cord stimulator. Frontal chest radiograph obtained before the procedure. The upper arrow indicates the ventriculoperitoneal shunt tubing descending over the right side of the neck and thorax. The middle and lower arrows indicate the cranial and caudal ends of the electrode contact arrays of the two percutaneous epidural leads, which lie in the midline over the lower thoracic spine at the T8-T11 levels. The leads continue caudally to the implantable pulse generator in the left lower back, below the field of view (Figure 5). No cardiac implantable electronic device is present. The patient's right is on the viewer's left; L, radiographic left side marker.

**Figure 5:**
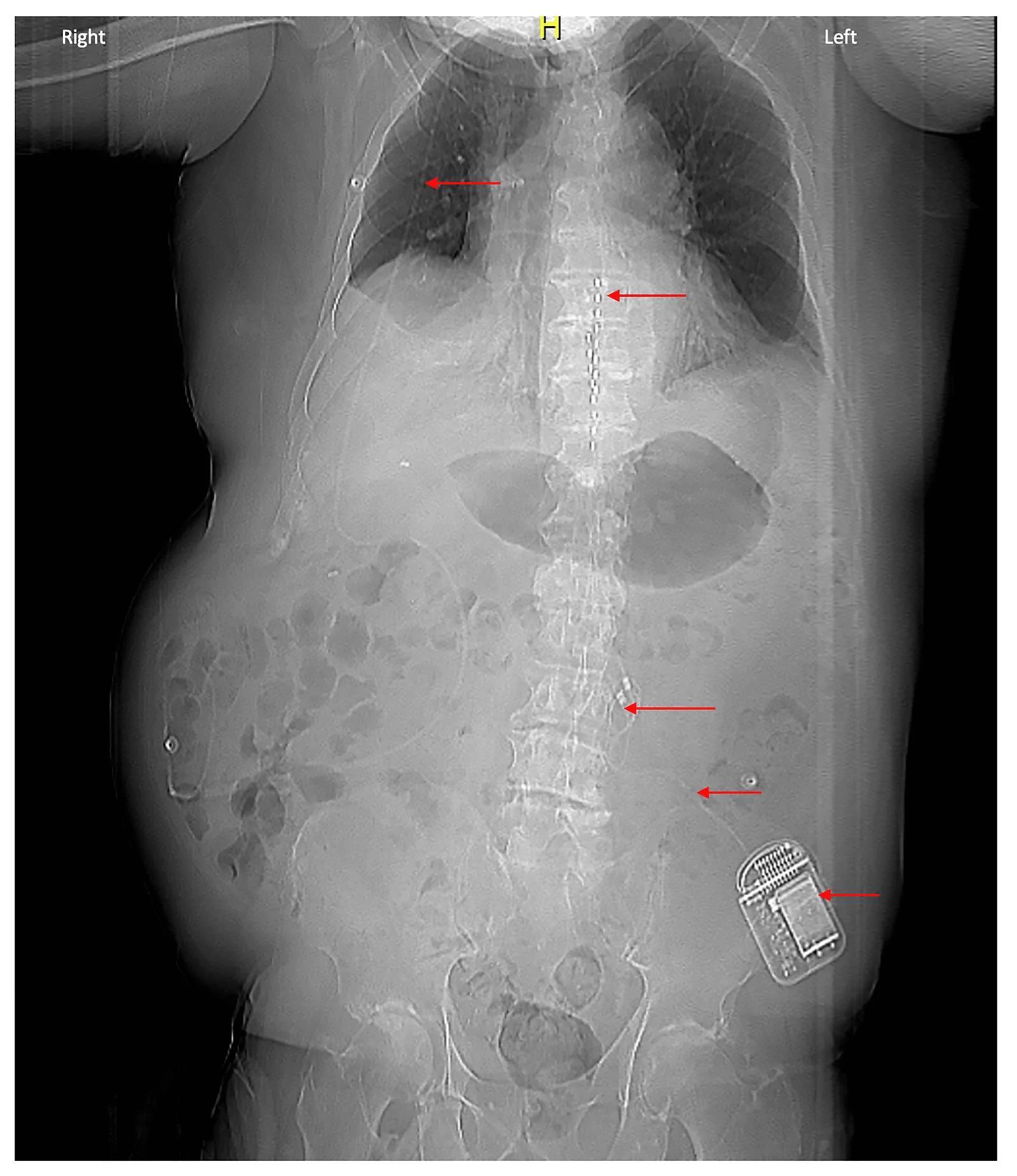
Frontal computed tomography scout image of the chest, abdomen, and pelvis showing the implantable pulse generator and the course of the leads. From top to bottom, the arrows indicate the ventriculoperitoneal shunt tubing; the electrode contacts of the epidural leads over the lower thoracic spine; the leads passing from the generator medially and cranially towards the spine (two arrows); and the implantable pulse generator (Nevro NIPG1500), a rounded rectangular radiopaque device projected over the left iliac region that corresponds to its subcutaneous pocket in the left lower back and upper gluteal region. The position of the generator, lateral to the spine and well below the diaphragm, informed the choice of a bipolar right ventricular pacing lead and the placement of the external defibrillator pads. The patient's right is on the viewer's left.

The technician recommended that the stimulator remain switched off for the duration of the procedure and that rapid ventricular pacing be delivered in a bipolar configuration; if unipolar pacing were required, the return (indifferent) electrode should be placed as far from, and on the opposite side to, the pulse generator as possible. The team elected to follow this advice. It was agreed in advance that the remote would be present in the catheterisation laboratory, that external defibrillator pads would be positioned away from the pulse generator and the course of the leads, and that the device would remain off if defibrillation, cardioversion, or conversion to open surgery became necessary.

Procedure

On the day of the procedure (day 43), the stimulator was switched off with the patient's own remote before he entered the catheterisation laboratory. He reported no clinically significant increase in pain, and the remote was kept immediately to hand in the procedure room. The procedure was performed in the cardiac catheterisation laboratory under conscious sedation and local anaesthesia. External defibrillator pads were applied in an anterolateral configuration, with the anterior pad in the right infraclavicular position and the lateral pad over the left mid-axillary line at the level of the cardiac apex. An anterior-posterior configuration was deliberately avoided because a posterior pad would have lain close to the generator pocket in the left lower back and the shock vector would have crossed the course of the leads; the anterolateral vector kept both pads and the current path cephalad to the generator. Vascular access comprised a 6-French sheath in the left femoral vein for a bipolar temporary pacing lead positioned in the right ventricle, a 7-French sheath in the left femoral artery for the pigtail catheter, and an 8-French sheath in the right femoral artery for valve delivery. Unfractionated heparin was administered to achieve therapeutic anticoagulation. Balloon aortic valvuloplasty was performed, and a 26 mm SAPIEN 3 Ultra RESILIA balloon-expandable valve (Edwards Lifesciences, Irvine, CA, USA) was deployed under bipolar rapid ventricular pacing at 180 beats per minute. No diathermy was used at any stage, and neither defibrillation nor cardioversion was required. Immediately after deployment, the electrocardiogram showed a new left bundle branch block, which was clearly interpretable in the absence of stimulator artefact. Post-deployment transthoracic echocardiographic assessment showed a mean transvalvular gradient of 12 mmHg with no valvular or paravalvular regurgitation. The patient remained comfortable throughout and did not require conversion to general anaesthesia.

Postprocedural course and follow-up

The patient was transferred to the cardiac care unit for continuous telemetry. The stimulator was switched on again by the patient with his own remote approximately six hours after the procedure, once he was settled in the cardiac care unit; therapy resumed at the preprocedural programme, and he reported that his back pain returned to its usual controlled level. The interval before reactivation was not dictated by the device or by the manufacturer. It reflected the period of supine bed rest and femoral access site surveillance after sheath removal, the initial period of close electrocardiographic observation of the new left bundle branch block, and transfer from the recovery area to the cardiac care unit, after which the patient was settled, had been reviewed by the anaesthetic team, and was able to operate the remote himself. During this interval, he required no supplementary analgesia beyond his regular oral paracetamol, and no opioid was administered. Transthoracic echocardiography on day 44 showed a well-seated prosthesis with trivial paravalvular regurgitation (Figure 6). In summary, the mean transvalvular gradient fell from 45 mmHg before the procedure to 9 mmHg on day 44 and the peak transaortic velocity from 4.5 to 1.9 m/s, and the Doppler velocity index rose from 0.22 to 0.83, with preserved left ventricular systolic function (ejection fraction 71%) and trivial paravalvular regurgitation. On the same day, telemetry detected a self-limiting episode of new-onset atrial fibrillation.

**Figure 6:**
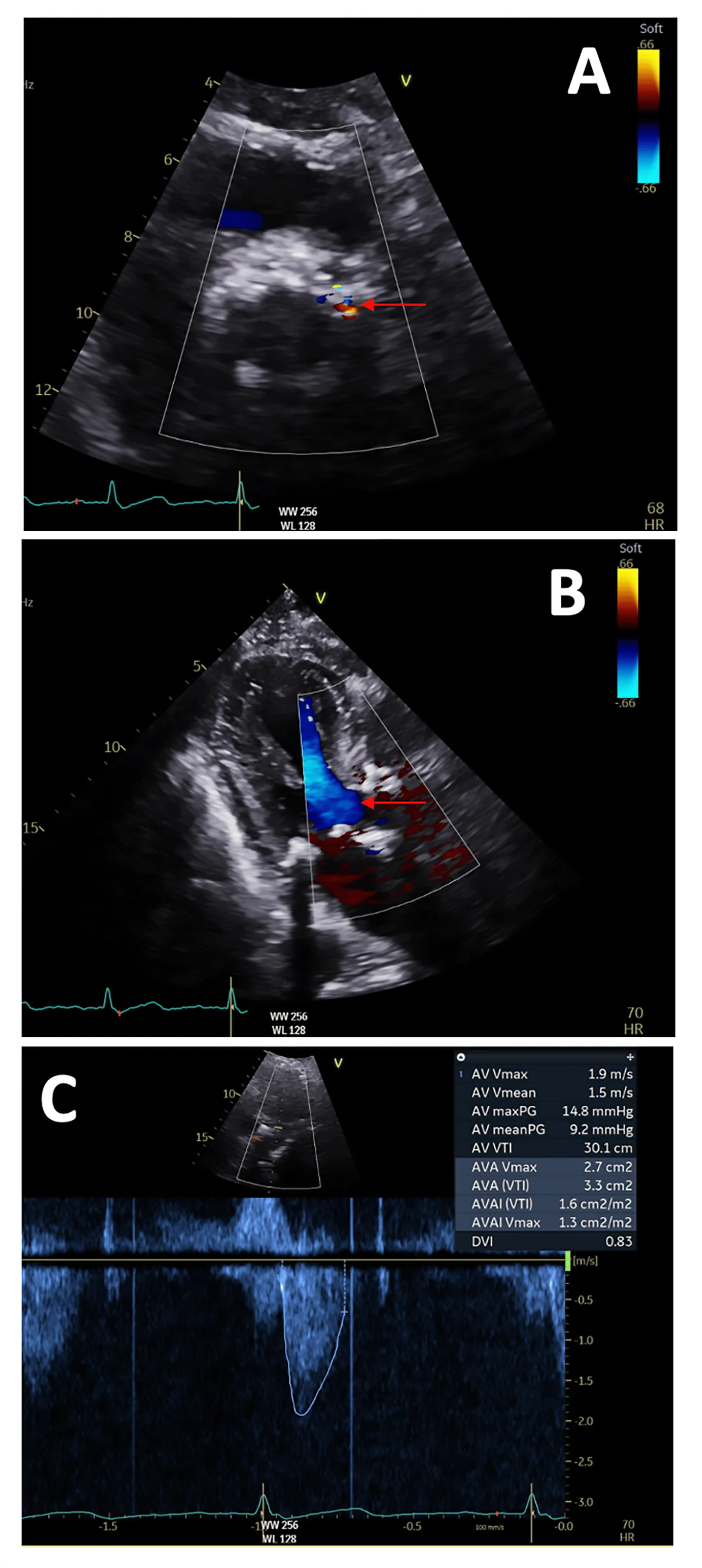
Transthoracic echocardiography on day 44, the day after the implantation of a 26 mm SAPIEN 3 Ultra RESILIA balloon-expandable transcatheter heart valve (Edwards Lifesciences, Irvine, CA, USA). (A) Parasternal short-axis view at the level of the aortic valve demonstrating the circular stent frame of the transcatheter heart valve, well expanded and appropriately seated within the native annulus. Colour flow Doppler (Nyquist limit 0.66 m/s) shows a small, localised colour signal adjacent to the stent frame at approximately the 4 o'clock position (arrow), consistent with trivial paravalvular regurgitation. (B) Apical five-chamber view with colour flow Doppler (Nyquist limit 0.66 m/s) demonstrating laminar antegrade systolic flow through the left ventricular outflow tract and across the transcatheter heart valve, without aliasing to suggest flow acceleration or obstruction. The arrow indicates the transcatheter heart valve. (C) Continuous-wave Doppler interrogation across the transcatheter heart valve from the apical window. The traced envelope shows a peak velocity of 1.9 m/s, mean velocity of 1.5 m/s, peak gradient of 14.8 mmHg, mean gradient of 9.2 mmHg, and velocity time integral of 30.1 cm. The effective orifice area was 3.3 cm² by the velocity time integral method and 2.7 cm² by the peak velocity method (indexed 1.6 cm²/m² and 1.3 cm²/m², respectively), with a Doppler velocity index of 0.83. These findings are consistent with normal transcatheter heart valve haemodynamics without prosthesis patient mismatch [24] and represent a substantial reduction from the preoperative mean gradient of 45.0 mmHg and peak velocity of 4.5 m/s (Figure 2B). The absolute effective orifice area derived by the continuity equation (3.3 cm² by the velocity time integral method) is higher than the normal reference values reported for a 26 mm SAPIEN 3 valve, which are in the order of 1.9 cm² [25]. After TAVI, the continuity equation is sensitive to the left ventricular outflow tract diameter entered, to whether that diameter is measured at the native annulus or at the inflow of the stent frame, and to the position of the pulsed-wave Doppler sample volume relative to the stent; an overestimated outflow tract area, or sampling within the region of flow acceleration at the stent inflow, will inflate the calculated area [24,25]. The value is reported as recorded on the clinical study and should be interpreted with this caveat. Prosthetic valve function was therefore characterised principally by the low mean gradient (9.2 mmHg) and the Doppler velocity index (0.83), both of which lie within the normal range for a transcatheter heart valve. AV: aortic valve; AVA: aortic valve area; AVAI: indexed aortic valve area; DVI: Doppler velocity index; HR: heart rate; maxPG: peak pressure gradient; meanPG: mean pressure gradient; Vmax: peak velocity; Vmean: mean velocity; VTI: velocity time integral; WL: window level; WW: window width

His CHA2DS2-VASc score [26] was 3 out of a maximum of 9 (age 75 years or older, 2 points; hypertension, 1 point). A score of 2 or more indicates elevated thromboembolic risk, for which oral anticoagulation is ordinarily recommended [27]. Anticoagulation was withheld after an individualised assessment of stroke and bleeding risk. Spontaneous termination of the arrhythmia does not of itself remove thromboembolic risk; the decisive consideration was the subdural haematoma six weeks earlier, which was judged to confer a risk of recurrent intracranial bleeding that outweighed the thromboembolic risk associated with a CHA2DS2-VASc score of 3. The decision was documented as provisional, to be revisited at outpatient review in light of the burden of atrial fibrillation on surveillance and the interval from the intracranial haemorrhage. The left bundle branch block resolved seven hours after the procedure; the patient's baseline first-degree atrioventricular block did not progress to high-grade block, and a permanent pacemaker was not required. He was transferred to his local hospital on day 45 on aspirin, with outpatient cardiology follow-up for surveillance of atrial fibrillation.

Interrogation by a Nevro device representative on day 78, five weeks after the procedure, confirmed no device issues and that the stimulator was functioning as intended. At telephone follow-up on day 85, six weeks after the procedure, the patient confirmed that the stimulator was functioning normally and that his back pain remained controlled at its preprocedural level. The sequence of events is summarised in Table 1.

**Table 1:** Timeline of clinical events. Day 1 is the day of admission to another hospital following the fall; earlier intervals are given relative to day 1. AVA: aortic valve area; ECG: electrocardiogram; LBBB: left bundle branch block; LVEF: left ventricular ejection fraction; TAVI: transcatheter aortic valve implantation; TTE: transthoracic echocardiography

Time point	Event
13 years earlier	Right ventriculoperitoneal shunt for hydrocephalus
8 years earlier	Spinal cord stimulator implanted for chronic back pain (Nevro Senza HFX system, NIPG1500 generator)
12 months earlier	Severe aortic stenosis diagnosed; asymptomatic
Day 1	Fall with subdural haematoma, admitted to another hospital; during that admission, 15-minute episode of chest pain at rest, peak high-sensitivity troponin 46 ng/L, and new ECG changes
Day 40	Transfer to our cardiology unit; TTE (mean gradient: 45 mmHg; AVA: 0.8 cm²; LVEF: 71%); heart team acceptance for transfemoral TAVI; discharge pending readmission
Day 42	Pre-anaesthetic assessment; implanting pain service technician contacted; device plan agreed
Day 43	Stimulator switched off with the patient's remote; transfemoral TAVI under sedation; 26 mm SAPIEN 3 Ultra RESILIA valve; bipolar rapid ventricular pacing at 180 beats per minute; new LBBB; transfer to the cardiac care unit
Day 43, after the procedure	Stimulator switched on with the patient's remote approximately six hours after the procedure; analgesia at baseline; LBBB resolved seven hours after the procedure
Day 44	TTE: well-seated prosthesis, trivial paravalvular regurgitation (Figure 6); self-limiting new-onset atrial fibrillation on telemetry; anticoagulation withheld
Day 45	Transferred to patient's local hospital on aspirin with outpatient cardiology follow-up
Day 78	Follow-up by Nevro device representative: device functioning as intended without any issues
Day 85	Telephone follow-up: device function and pain control confirmed

## Discussion

This report describes the periprocedural management of a high-frequency spinal cord stimulator in an octogenarian undergoing transfemoral TAVI under sedation, a combination for which we found only one brief previous mention [20]. The stimulator was switched off with the patient's own remote, which stayed in the room; rapid ventricular pacing was delivered through a bipolar right ventricular lead; and external defibrillator pads were positioned away from the pulse generator and the course of the leads. The procedure was uneventful. The patient tolerated several hours without stimulation with no clinically significant increase in pain; the surface electrocardiogram was free of stimulator artefact when a new left bundle branch block appeared immediately after valve deployment, stimulation was resumed at the preprocedural programme with analgesia equivalent to baseline, and interrogation by the manufacturer's representative five weeks after the procedure confirmed normal device function. It should be stated plainly that the case demonstrates the successful application of a precautionary strategy. Because the stimulator was off throughout, no direct observation was made of an active spinal cord stimulator during rapid ventricular pacing, and the case cannot show that any individual precaution was necessary or establish the comparative safety of the alternatives. The value of the case lies less in the outcome than in the reasoning, because the hazards that existing guidance is designed to control are not those that the catheterisation laboratory presents.

Why theatre-based guidance transfers imperfectly

The published recommendations for patients with a spinal cord stimulator undergoing surgery are consistent and well founded [5,10-12,28-30]. They address four hazards: damage to the pulse generator or heating of the leads by external electromagnetic energy, inadvertent or altered stimulation causing discomfort or neural injury, lead migration during positioning, and degradation of the electrocardiogram by stimulation artefact [4,10,28,29]. The manufacturers' labelling on which those recommendations draw is written around monopolar electrosurgery, defibrillation, cardioversion, lithotripsy, and magnetic resonance imaging [10,29,31,32]. During an uncomplicated transfemoral TAVI, none of these is used. What the procedure does involve, and what the theatre literature does not consider, is a temporary transvenous or guidewire pacing system delivering rapid ventricular pacing while the patient is awake, together with a small but well-documented probability that defibrillation, cardioversion, or emergent sternotomy will be needed at short notice [13-15]. The anaesthetist is therefore obliged to reason from first principles and from analogous evidence. The considerations that shaped our plan are examined in turn below and summarised, with an indication of the evidence on which each recommendation rests.

Rapid ventricular pacing

Rapid ventricular pacing at 160-220 beats per minute is used during balloon valvuloplasty and valve deployment to abolish transvalvular flow for a few seconds. It is delivered through a transvenous bipolar right ventricular lead or, increasingly, through the left ventricular delivery guidewire in a unipolar configuration with a return electrode placed subcutaneously at the femoral access site or on a second intravascular wire [13]. In a bipolar circuit, the current path is confined to a few millimetres of myocardium between the two lead electrodes. In a unipolar circuit, the path runs from the endocardial cathode through the thorax or abdomen to the return electrode, and its geometry depends entirely on where that electrode is placed. In our patient, whose pulse generator lay in the left lower back and gluteal region, a groin return electrode would have brought the current path close to the generator pocket, and this was the specific reason for preferring a bipolar right ventricular lead, a choice that the implanting service had also recommended. Where a unipolar guidewire technique is preferred in a patient whose generator lies in the lower back or gluteal region, the return electrode should be positioned cephalad to, and on the side opposite, the generator, for example, as a skin electrode over the contralateral shoulder or upper arm or by using a second wire or sheath in the contralateral upper limb (radial or brachial) as the indifferent electrode, so that the current path between the left ventricular cathode and the return electrode remains above the diaphragm and remote from the generator pocket and the course of the leads. A femoral or groin return electrode should be avoided in this situation. This is an extrapolation from the principles that govern return electrode placement for monopolar electrosurgery [10,28] rather than a validated recommendation.

The energy involved is, however, very small. A temporary pacing stimulus of a few milliamperes lasting 1-2 ms across a lead impedance of a few hundred ohms delivers in the order of tens to a few hundred microjoules per pulse [33], whereas a single external biphasic shock delivers 150 J or more [34], a difference of some six orders of magnitude. Manufacturers' labelling does not list cardiac pacing among the sources of electromagnetic interference [31,32]. The most relevant clinical evidence comes from patients in whom a spinal cord stimulator and a permanent pacemaker or defibrillator coexist for years. In 18 patients with a thoracic spinal cord stimulator for refractory angina and a permanent pacemaker, stimulation at the maximum tolerated amplitude with pacemaker sensitivity deliberately increased produced no inhibition of pacing [35]; five patients with a spinal cord stimulator and an implantable cardioverter-defibrillator experienced no inappropriate shocks [36]; and two systematic reviews of the coexistence literature identified interference in only a small minority of cases, almost always in the direction of the stimulator affecting cardiac device sensing rather than the reverse [18,19]. Sensing is irrelevant to a temporary asynchronous pacing run, and the reverse interaction, a pacing stimulus damaging or reprogramming a spinal cord stimulator, has not been described. We therefore regard clinically significant interaction between rapid ventricular pacing and a spinal cord stimulator as improbable while recognising that neither the disparity in energy nor the absence of cardiac pacing from manufacturers' lists of interference sources establishes that interference cannot occur. A bipolar configuration confines the current path and reduces the theoretical concern at no cost, but it does not eliminate it, and its protective value has not been demonstrated. Our patient's device was off during the pacing runs and was functioning normally at interrogation five weeks later, which is consistent with this expectation but does not test it.

Defibrillation and cardioversion

External defibrillation is not part of a planned TAVI but must be anticipated. In a single-centre series of 2,102 procedures, 51 patients (2.5%) suffered a catastrophic intraprocedural cardiac event; among the 10 who developed acute left ventricular failure at deployment, six with ventricular fibrillation or sustained ventricular tachycardia were defibrillated, and four had pulseless electrical activity [14]. In a smaller European series, 59 of 729 patients required intraprocedural cardiopulmonary resuscitation, with in-hospital mortality of 17% [37]. New-onset atrial fibrillation follows TAVI in 8.4% of patients overall and 4.4% of transfemoral cases, so that synchronised cardioversion may also be required in the days after the procedure [38]. Our patient developed exactly this complication on the day after the procedure. The arrhythmia terminated spontaneously, and cardioversion was not needed, but the possibility had been discussed in advance, and it had been agreed that the stimulator would remain off if a shock became necessary.

The manufacturers are candid that the safety of external defibrillation in a patient with a spinal cord stimulator has not been established, and one warns that a shock may induce currents in the lead extension [31]. The risk is not hypothetical: in June 2023, a field safety notice reported that cardioversion had rendered two implantable neurostimulators of one model unresponsive, both requiring explantation, and recommended that before planned cardioversion, the device be temporarily reprogrammed to the settings specified in its labelling rather than simply switched off [39]. That advice is model-specific and illustrates why the current manual, rather than a generic rule, must be consulted (Table 2). Three principles nevertheless apply to every device. First, the presence of a spinal cord stimulator must never delay defibrillation of a shockable rhythm [10,40]; time-critical reprogramming is not realistic during cardiac arrest, and the stimulator should simply be off. Second, because pads are applied routinely before every TAVI, their position can be chosen deliberately: as far from the pulse generator and leads as practicable, with the shock vector orientated so that it does not pass through the system, using an anterior-posterior configuration only if the posterior pad can be kept well clear of the generator [10,11]. In our patient, whose generator lay in the left lower back, an anterolateral configuration (right infraclavicular to left mid-axillary) kept both pads and the shock vector cephalad to the generator pocket and clear of the course of the leads. Third, if a shock is delivered, the device should be interrogated afterwards and the implanting service informed, since damage may not be apparent to the patient until therapy is next required [10,31,39].

**Table 2:** Manufacturer-specific considerations for spinal cord stimulation systems in common. Systems listed are those in common use at the time of writing. Recommendations for Abbott and Nevro systems and the Medtronic Vanta advisory are drawn from manufacturer labelling and safety notices [31,32,39]; those for other systems reflect published perioperative guidance [10,12]. The current labelling for the specific model should always be consulted, because instructions differ between models and change over time.

Manufacturer	Current systems	Perioperative considerations
Abbott	Eterna; Proclaim XR, Plus and Elite; Proclaim DRG	Abbott devices have a dedicated Surgery Mode that suspends stimulation and is recommended in preference to switching the device fully off [31]. Enter Surgery Mode with the patient controller before the procedure, exit it afterwards, and then confirm device function. The manufacturer advises bipolar electrosurgery only, states that the safety of external defibrillation has not been established, and asks that the system be checked after any defibrillation [31]
Boston Scientific	WaveWriter Alpha and Alpha Prime; Precision Montage, Novi and Spectra	Switch off before the procedure and on afterwards in accordance with the current manufacturer instructions
Medtronic	Intellis; Vanta; Sequentia LT; Inceptiv	Switch off before the procedure and on afterwards in accordance with the current manufacturer instructions. A June 2023 field safety notice reported two Vanta (model 977006) generators rendered unresponsive by cardioversion, both requiring explantation, and recommended temporary reprogramming to the settings specified in the current labelling before planned cardioversion rather than switching the device off [39]. Consult the current labelling for the recommended settings and for the interval between shocks
Nevro	Senza; Senza II; Senza Omnia (HFX)	Switch off before the procedure and on afterwards in accordance with the current manufacturer instructions [32]. Because 10 kHz therapy is paraesthesia-free, confirm the device state from the remote control rather than from the patient's symptoms
Saluda Medical	Evoke	Switch off before the procedure and on afterwards in accordance with the current manufacturer instructions. During a trial period, disconnect the external trial stimulator from the percutaneous leads before the procedure

Emergent surgery and diathermy

Conversion to emergent cardiac surgery occurred in 0.76% of 27,760 transfemoral procedures in the European EuRECS-TAVI registry, most often for annular rupture, ventricular perforation, coronary obstruction, or device embolisation [15], and in 11 of the 2,102 patients in the series cited above, with in-hospital mortality of 54.5% [14]. In a national inpatient sample of 383,395 TAVI admissions between 2016 and 2021, 1.2% involved a major complication requiring surgical or mechanical circulatory rescue, and although the proportion has been stable, the absolute number is rising with procedural volume [41]. In these circumstances, a sedated patient is anaesthetised and intubated, the chest is opened, and monopolar diathermy is used at high power, often without time to reconsider the neuromodulation system. Diathermy current can be conducted along the leads to the epidural electrodes, causing thermal injury to neural tissue, and can damage the generator whether or not it is switched on [4,10,29,32]. The recommendations for theatre therefore apply in full: the stimulator must be off; bipolar diathermy is preferred; where monopolar diathermy is unavoidable, the return plate is placed as far from the system as possible and on the side opposite the generator, so that the current path between the active electrode and the plate does not cross the generator, extension, or leads; the lowest effective power is used; and the device is interrogated afterwards [10,28,29]. The important practical point is that these decisions cannot be made calmly once conversion has begun. In our patient, they were settled in advance: the position of the generator and leads was known to the team before the procedure started, and it had been agreed that the device would remain off if conversion became necessary.

Electrocardiographic surveillance and conduction

Continuous electrocardiographic monitoring is of greater importance during TAVI than during most non-cardiac surgery. Capture must be confirmed during each pacing run, coronary obstruction declares itself through ST-segment change, and new conduction disturbance is the commonest complication of the procedure, with new left bundle branch block in a substantial proportion of balloon-expandable implants and permanent pacing required in roughly one patient in 10 [7,16]. The management of these disturbances rests on serial electrocardiographic interpretation over the following 24-48 hours [42]. Active neurostimulators are a recognised source of electrocardiographic artefact: in a systematic evaluation of 4,096 leads in patients with deep brain stimulators, monopolar stimulation interfered with rhythm and frequency interpretation significantly more often than bipolar stimulation, and interference increased with stimulation voltage [17]. Comparable data for SCS are limited to case reports, and modern high-frequency systems may behave differently from conventional devices, but the mechanism is the same. In our patient, who entered the procedure with first-degree atrioventricular block and developed a new left bundle branch block at valve deployment and then new atrial fibrillation on telemetry the following day, unambiguous electrocardiography was not a convenience but a safety requirement: each of these findings had a management consequence, from the assessment of the need for permanent pacing to the withholding of anticoagulation. Switching the stimulator off removed the question of artefact entirely.

A further feature of high-frequency stimulation is relevant here. Because 10 kHz therapy is paraesthesia-free [43], the patient cannot tell from sensation whether the device is on or off, and the state of the device must be confirmed from the remote rather than inferred from symptoms. The delayed offset of analgesia that is characteristic of high-frequency stimulation may also explain why our patient tolerated several hours without therapy comfortably, although we did not measure this formally and it should not be assumed for conventional paraesthesia-based systems.

Switching off or leaving on

Leaving a spinal cord stimulator active during TAVI has not been evaluated, in this case or elsewhere, and we do not present it as a generally acceptable alternative. The absence of a planned source of high-energy interference does not of itself make continued stimulation safe, because defibrillation, cardioversion, and conversion to surgery can be required without warning. There may nevertheless be a patient under sedation whose pain without stimulation would make lying still for one to two hours intolerable and in whom the balance of risks favours continued stimulation. If that course is contemplated, it should be a deliberate and documented decision reached with the implanting service and the structural heart team and informed by the current labelling for the specific model, since some manufacturers specify a surgical mode or particular settings rather than simple deactivation. A baseline electrocardiogram should be recorded with stimulation active to confirm that the trace is interpretable, the remote must be in the room with a named person responsible for it, and the device must be switched off immediately if defibrillation, cardioversion, or conversion to surgery becomes necessary [10,12]. We nevertheless consider deactivation, at the lowest amplitude and with the remote to hand, to be the correct default, for three reasons: it removes the possibility of artefact at the moments when the electrocardiogram matters most; it eliminates a step from the sequence of actions required in a time-critical emergency, in which the person holding the remote may also be the person managing the airway; and it aligns the catheterisation laboratory with the theatre convention that every member of the team already knows, so that nothing needs to be remembered under pressure. The awake patient is an ally rather than an obstacle in this approach: the patient can operate the remote, can confirm the device state and report any unexpected stimulation, and can be told before the procedure that an increase in pain during the case will be treated and is not a reason to switch the device back on unilaterally. Our patient had been told beforehand what to expect; any discomfort was minimal, and his usual analgesia returned once the device was switched on again.

Positioning and other implanted devices

Lead migration is the commonest hardware complication of SCS and is most likely in the first weeks after implantation, before fibrous anchoring is complete [28,30]. TAVI is performed supine on a flat table with little spinal movement, so the risk is smaller than for lateral or prone surgery, but transfers to and from the table should be performed with the spine in neutral alignment, the generator site should be identified and padded, and particular care is warranted when the system is less than six weeks old [10,28]. Our patient's system had been in place for eight years, so migration was not a material concern, although the position of the generator, confirmed radiographically beforehand, was known to the team when he was positioned. He also had a ventriculoperitoneal shunt. Although our patient had a fixed-pressure shunt valve, other patients may have programmable shunt valves that can be reset by static magnetic fields as low as 24-42 mT [44], and both the remote controls and the inductive chargers of rechargeable neurostimulators generate magnetic fields; the valve type should therefore be established at the pre-anaesthetic assessment and, if programmable, its setting checked after any period of charger use. Patients with several implanted devices are increasingly common, and each device changes the constraints on the others.

Postprocedural device management

After an uncomplicated procedure, the stimulator can be switched back on as soon as the patient is settled in the recovery area or cardiac care unit, and formal interrogation can await the next scheduled review [10]. This was the course followed in our patient, whose therapy was resumed at the preprocedural programme and whose device was functioning normally at routine interrogation. That position deserves qualification for a paraesthesia-free system, in which a silent reset, a change of programme, or a shift in lead impedance may produce no symptom that the patient can report and in which the return of analgesia is a delayed and imprecise indicator of normal function. Where a remote system check or an in-hospital interrogation by the implanting service or the device representative can be arranged before discharge, we would now regard it as advisable after any TAVI, and we consider in-hospital interrogation before discharge to be mandatory in the following circumstances: any exposure to defibrillation, cardioversion, or diathermy; intraprocedural haemodynamic instability or cardiac arrest; unexpected stimulation, paraesthesia, or loss of analgesia after reactivation; inability of the patient to confirm the device state or to operate the remote; and, in a patient with new conduction disturbance or arrhythmia under surveillance, before any planned cardioversion or pacemaker implantation. In our patient, the first formal interrogation took place at routine follow-up five weeks after the procedure; in retrospect, an earlier check would have been preferable and would have been capable of detecting a transient change that our approach could not. After any exposure to defibrillation, cardioversion, or diathermy, interrogation should be prompt, impedances and programming should be compared with the preprocedural record, and the implanting service should be told what occurred. The final device state and any energy exposure should be documented in the anaesthetic record and in the discharge summary. The point is not academic in a patient such as ours, who left hospital with first-degree atrioventricular block and a recent episode of atrial fibrillation under surveillance: later cardioversion, or pacemaker implantation for progressive conduction disease, may be undertaken by clinicians who would not otherwise know that a spinal cord stimulator is present. This is the approach that has been formalised for cardiac implantable electronic devices in the perioperative period [40], and the analogy is instructive: a written prescription for the device, agreed in advance with the implanting team and reversed after the procedure, replaced ad hoc decision-making in that field and could do so here.

A structured approach

The reasoning above can be reduced to six steps, which are summarised in Table 3 and, for the devices in common use, in Table 2. In Table 3, each recommendation is labelled according to its evidence basis: manufacturer labelling or safety notice (M), extrapolation from published perioperative guidance written for the operating theatre (E), or the authors' opinion derived from first principles and analogous evidence (O). None of these recommendations has been validated in the TAVI setting, and the approach is offered as a framework for individualised, multidisciplinary planning that must defer to the current instructions for the specific device, rather than as a protocol.

**Table 3:** Stepwise approach to the management of a spinal cord stimulator during TAVI. The table summarises the six-step approach described in the text, arranged by phase (preprocedural, intraprocedural, and postprocedural). The first section lists the three sources of electrical energy relevant to TAVI and their significance for an implanted stimulator. Switching the stimulator off is the default (Option A); leaving it on (Option B) is an unvalidated option, not evaluated in this case, that may be considered only for the patient who cannot otherwise tolerate the procedure and is conditional on an artefact-free baseline electrocardiogram and the ability to switch the device off without delay. The emergency section applies whichever option is chosen. Manufacturer-specific considerations, including Abbott Surgery Mode and the Medtronic Vanta cardioversion advisory, are summarised in Table 2. Evidence basis of each recommendation: M: manufacturer labelling or safety notice; E: extrapolated from published perioperative guidance written for the operating theatre; O: authors' opinion derived from first principles and analogous evidence. No recommendation has been validated in the TAVI setting, and the current labelling for the specific device takes precedence. TAVI: transcatheter aortic valve implantation The table was created by the authors from references [5,10-15,28-41,43].

Stages or consideration	Management and precautions
Sources of electrical energy relevant to an implanted stimulator during TAVI	
Rapid ventricular pacing	Used routinely during valvuloplasty and valve deployment. Each pulse delivers energy in the order of microjoules, and cardiac pacing is not listed by manufacturers as a source of interference. Clinically significant interaction is considered improbable; a bipolar configuration reduces, but does not eliminate, the theoretical concern (O)
Defibrillation or cardioversion	Not planned, but required for intraprocedural arrest or unstable arrhythmia, which complicate about 2.5-8% of procedures, and after the procedure for new atrial fibrillation. High-energy shocks can damage the generator or leads and injure the patient
Emergent surgery with diathermy	Not planned, but conversion to open surgery occurs in under 1% of transfemoral procedures. Monopolar current can be conducted along the leads, heating neural tissue and damaging the generator
Preprocedural spinal cord stimulator identified at the pre-anaesthetic assessment	
Step 1: identify the device	Document: manufacturer and model; indication; date of implantation; lead type and vertebral level; position of the pulse generator. Plain radiography locates the components if there is any doubt. Ask the patient to bring the remote control to the assessment and on the day of the procedure. Record the contact details of the implanting service and the device representative (E)
Step 2: consult the current manufacturer instructions and the implanting service	Instructions differ between manufacturers and change over time: obtain the current labelling for the specific model and contact the device representative if clarification is needed. Confirm how the device is switched off and on and whether any model-specific precaution applies to cardioversion. Manufacturer-specific considerations are summarised in Table 2 (M, E)
Step 3: decide the device state	
Option A (default): switch the stimulator off	Preferred: removes electrocardiographic artefact and simplifies the response to emergency defibrillation, cardioversion, or diathermy. Reprogram to the lowest amplitude, and then switch off with the remote before the patient enters the procedure room (Surgery Mode for Abbott devices). Confirm the device state from the remote rather than from symptoms: 10 kHz therapy is paraesthesia-free (E; Surgery Mode, M)
Option B: leave the stimulator on	Consider only when pain without stimulation would make lying still intolerable, after discussion with the implanting service and review of the current labelling for the specific model. An unvalidated option, not evaluated in this case (O): the absence of planned defibrillation, cardioversion, or diathermy does not of itself make continued stimulation safe. Record a baseline electrocardiogram with therapy on; if artefact is present, switch the device off and manage pain by other means. Adds a step to the emergency sequence: the device must be switched off at once if defibrillation, cardioversion, or diathermy is needed
Step 4: prepare the procedure room	Keep the remote control in the room, with a named person able to switch the device off without delay. Transfer and position the patient with the spine in neutral alignment; identify and pad the generator site; take particular care if the system is less than six weeks old. Place external defibrillator pads as far from the generator and leads as practicable, so that the shock vector does not pass through the system. For a generator in the lower back or gluteal region, an anterolateral configuration is usually appropriate; avoid an anterior-posterior configuration unless the posterior pad can be kept well clear of the generator (E). Brief the team, including the surgeon and perfusionist on standby, on the position of the generator and leads (O)
Intraprocedural	
Step 5: during the procedure	Confirm the device state before rapid ventricular pacing. A bipolar right ventricular lead is preferred; if a unipolar guidewire technique is used, place the return electrode cephalad to and on the side opposite the generator (for example, over the contralateral shoulder or upper arm or via a contralateral upper limb sheath) so that the current path remains above the diaphragm and remote from the generator and leads; avoid a groin return electrode when the generator is in the lower back or gluteal region (E, O). Unexpected artefact or paraesthesia: confirm that the stimulator is off (or in Surgery Mode for Abbott devices)
Emergency defibrillation, cardioversion, or conversion to surgery with diathermy	
Defibrillation or cardioversion	Never delay defibrillation of a shockable rhythm because a stimulator is present. If the stimulator was left on, switch it off immediately. Use the pad positions chosen in Step 4 and the lowest clinically appropriate energy. Model-specific reprogramming for planned cardioversion (for example, Medtronic Vanta) is unlikely to be practicable in an unstable patient (M, E)
Diathermy	The stimulator must be off; use bipolar diathermy wherever possible. If monopolar diathermy is unavoidable, place the return electrode as far from the system as possible and on the side opposite the generator, so that the current path does not cross the generator or leads. Use the lowest effective output and interrogate the device afterwards (M, E)
Postprocedural	
Step 6: after the procedure	Switch the stimulator on (exit Surgery Mode for Abbott devices) once the patient is settled, and confirm that analgesia has returned. After exposure to defibrillation, cardioversion, or diathermy: interrogate promptly, compare impedances and programming with the preprocedural record, and inform the implanting service. After an uncomplicated procedure, arrange a remote system check or in-hospital interrogation before discharge where available, particularly for a paraesthesia-free system (O). In-hospital interrogation before discharge is mandatory after any energy exposure, intraprocedural instability or arrest, unexpected stimulation or loss of analgesia, or inability of the patient to confirm the device state and before any planned cardioversion or pacemaker implantation (O). Record the final device state and any energy exposure in the anaesthetic record and in the discharge summary

Limitations

This is a single case, and it cannot establish safety. Because the stimulator was off, our report provides no information on the behaviour of an active spinal cord stimulator during rapid ventricular pacing, and the bipolar configuration means that the unipolar guidewire technique was not tested. Device impedances were not measured immediately before and after the procedure; the first formal interrogation took place at routine follow-up, so a transient change would not have been detected. Our recommendations for the specific device are drawn from its labelling and from the implanting service rather than from measurement. The case therefore cannot establish the necessity or the comparative safety of any individual component of the strategy, and the option of leaving a stimulator active, which we discuss, was not evaluated. The postprocedural effective orifice area recorded on the clinical echocardiogram was higher than expected for the valve size, most plausibly because of the sensitivity of the continuity equation to outflow tract measurement after TAVI; the low gradient and normal Doppler velocity index are the more reliable indices of prosthetic function in this setting. The approach we describe is one of risk avoidance, and the questions it raises, in particular whether electrocardiographic artefact from a modern high-frequency SCS is clinically material and how an active stimulator behaves during a unipolar pacing run, are answerable only by systematic observation across centres. We hope this report prompts that work.

## Conclusions

Patients with a spinal cord stimulator will increasingly present for TAVI, and the guidance written for the operating theatre does not describe the exposures they will meet. Rapid ventricular pacing carries a low theoretical risk of interaction that a bipolar configuration is expected to reduce, whereas defibrillation, cardioversion, and conversion to surgery, although uncommon, are the exposures that can damage a device and must be planned for before the procedure begins. Identifying the device and its manufacturer, consulting the current labelling and the implanting service, switching the stimulator off with the remote to hand, positioning defibrillator pads with the generator and leads in mind, and confirming device function afterwards were the components of a precautionary strategy with which this procedure was completed under sedation without observed stimulator malfunction. A single case cannot establish that any of these measures was necessary nor demonstrate their comparative safety. Leaving the stimulator active is a conditional and unvalidated option, not supported by observations in this case, that may be considered after multidisciplinary discussion for the patient who cannot otherwise tolerate the procedure, provided the current labelling for the specific model permits it, the electrocardiogram is confirmed to be interpretable, and the device can be deactivated without delay.
